# Curiosity-driven search for novel nonequilibrium behaviors

**DOI:** 10.1103/physrevresearch.6.033052

**Published:** 2024-07-11

**Authors:** Martin J. Falk, Finnegan D. Roach, William Gilpin, Arvind Murugan

**Affiliations:** 1 Department of Physics, The University of Chicago, Chicago, Illinois 60637, USA; 2 Department of Physics, The University of Texas, Austin, Texas 78712, USA

## Abstract

Exploring the full spectrum of novel behaviors that a system can produce can be an intensive task. Sampling techniques developed in response to this exploration challenge often require a predefined metric, such as distance in a space of known order parameters. However, order parameters are rarely known for nonequilibrium systems, especially in the absence of a diverse set of example behaviors, creating a chicken-and-egg problem. Here, we combine active and unsupervised learning for automated exploration of nonequilibrium systems with unknown order parameters. We iteratively use active learning based on current order parameters to expand the library of behaviors and relearn order parameters based on this expanded library. We demonstrate the utility of this approach in Kuramoto models of increasing complexity. In addition to reproducing known phases, we reveal previously unknown behavior and related order parameters, and we demonstrate how to align search with human intuition.

## I. INTRODUCTION

One of the first exciting things to do with a new physical system is to go exploring—to tune parameters and see what unexpected behaviors the system is capable of. Through open-ended exploration, we build intuition for the right variables to describe the system, which can subsequently be used for more systematic investigation.

In many contexts, for example in driven materials with space- and time-dependent activity [1–6], parameter spaces are growing increasingly high-dimensional, and the output behaviors increasingly complex. Methods to automate exploration of such novel high-dimensional systems, including active learning [7–9], evolutionary searches [10,11], and Bayesian optimization [12–15], require predefined metrics, such as distances in a space of known order parameters, to characterize system behavior. However, methods to construct order parameters typically assume data sets with sufficient diversity of behaviors [16–23], which are difficult to gather without using automated exploration methods.

This leads to a chicken-and-egg problem; exploring behaviors in high-dimensional systems requires good order parameters, but finding good order parameters means observing behaviors of sufficient diversity. If we choose bad order parameters, we potentially miss rich behaviors that were not anticipated by the metric choice, a particular concern in nonequilibrium many-body systems with generically unknown order parameters [23–26].

Is it possible to perform automated exploration of high-dimensional parameter spaces without assumptions on what the order parameters are for an interesting output behavior? Data-driven dimensionality reduction techniques and, separately, active learning have addressed components of that challenge, but they are individually not sufficient to tackle the challenge as a whole.

Here, we demonstrate how a curiosity-driven search algorithm can efficiently explore nonequilibrium many-body systems, even in the absence of previously known order parameters. We adapt methods that combine the strengths of both active learning and dimensionality reduction [27–30]. We learn order parameters through unsupervised dimensionality reduction on a library of currently known behaviors; we then sample in the space of current order parameters to reveal new behaviors and iterate. Crucially, we search in the learned low-dimensional latent space trained on dynamical behaviors rather than the high-dimensional parameter space; in this way, active learning efficiently samples richer parts of parameter space.

We apply this framework to a paradigmatic class of dynamical systems—the Kuramoto model of coupled oscillators and its variants [31,32]. We first use curiosity search to benchmark against known results on simple Kuramoto model variants with one or two parameters, which are nevertheless capable of producing rich nonequilibrium behaviors. We then explore a three-population Kuramoto model with 10 adjustable parameters, and we reveal previously uncharacterized behavior and corresponding order parameters. Finally, we demonstrate how curiosity search can be formulated to naturally align with human intuition in order to target multipopulation behaviors in a 10-population Kuramoto model with 100 parameters.

## II. METHOD

**FIG. 1.**
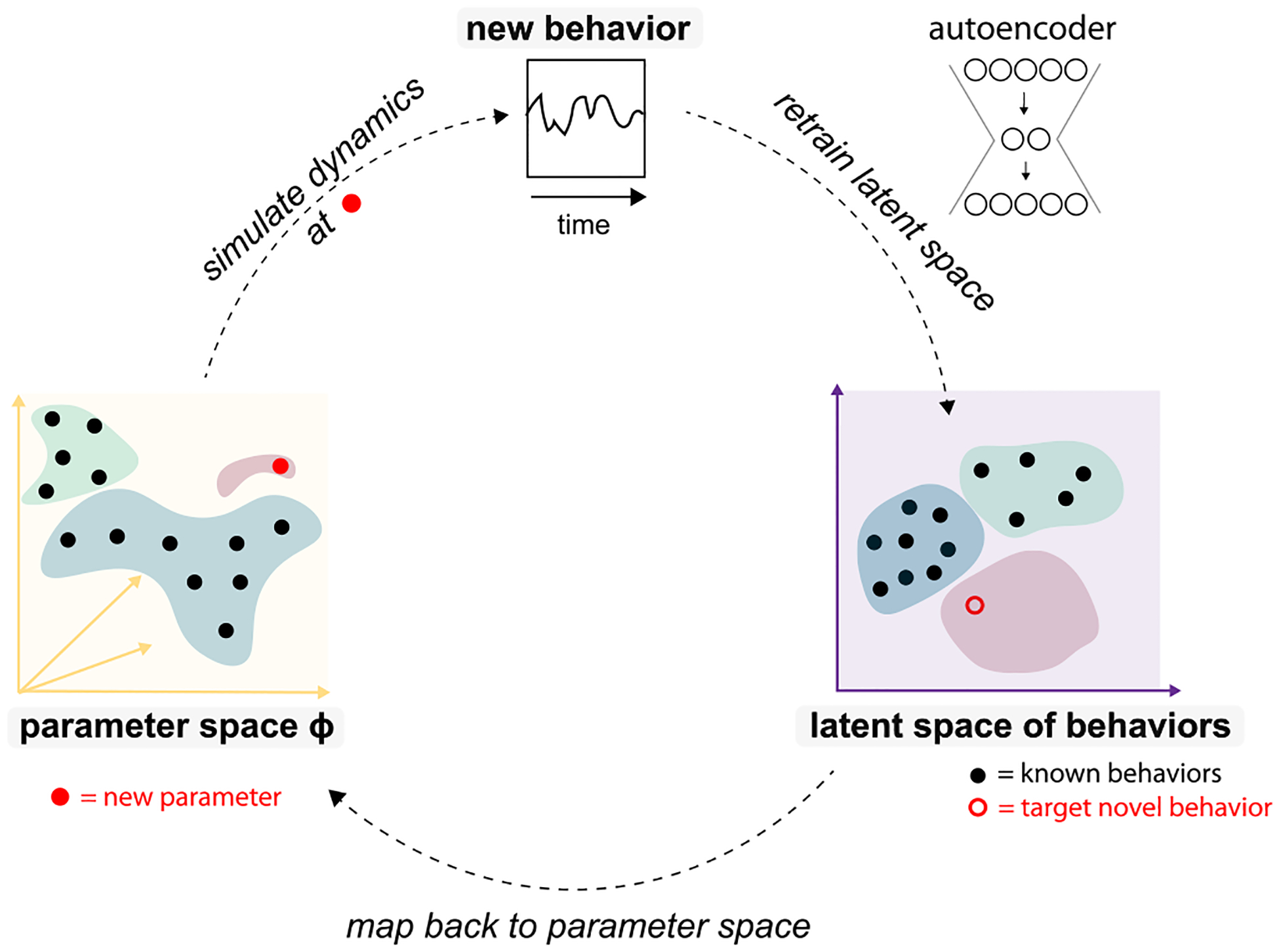
Overview of the curiosity-driven search for novel behaviors. We consider a system with a high-dimensional parameter space (yellow) whose potential behaviors and order parameters are initially unknown. Search is initialized by collecting behaviors corresponding to a uniform sampling of parameter space. These dynamical behaviors are used to train an autoencoder to obtain a low-dimensional latent space of behaviors (purple) parametrized by putative order parameters. We then seek a new behavior by randomly sampling the learned latent space (open red circle). We map the target new latent space point back to parameter space (solid red circle), evaluate the resulting behavior, and thus expand our library of known behaviors. Autoencoder is retrained every K training rounds on a random subset of previously sampled behaviors, thus improving the learned latent space and order parameters. (Green, purple, and blue regions of parameter and latent spaces indicate qualitatively distinct behaviors.)

The curiosity sampling algorithm has three key components shown in Fig. 1: a high-dimensional parameter space, a high-dimensional space of raw system behaviors, and a low-dimensional latent space of behaviors whose axes function as order parameters.

We begin from a small library of randomly sampled parameters and their associated dynamical behaviors. We then train a dimensionality reduction method on the assembled library of behaviors, creating a latent space characterizing those behaviors. Next, we search for new target behaviors in this emergent latent space of behaviors created by dimensionality reduction. The new target behaviors are mapped back to new points in parameter space to sample. We evaluate the behaviors at these parameters, thereby expanding our library of known behaviors. Finally, we retrain the dimensionality reduction so the latent space can incorporate information from the expanded library of behaviors, and we begin the whole cycle again.

As the cycles of exploration and dimensionality reduction repeat, more behaviors are observed, and the latent space becomes a more refined and accurate representation of those behaviors.

The protocol outlined above has several choices in the details of how different steps are implemented for a given system. For example, in the Kuramoto models we will study here, each behavior takes the form of N time series of oscillator phase with M subsampled time steps, where N is the number of oscillators. For each subsampled time, we construct the phase space density across the N oscillators and mean-center it, thereby accounting for permutation invariance and global mean rotations.

For dimensionality reduction, we use a convolutional variational autoencoder [33] (VAE) with relatively simple encoder and decoder architectures. In part to guard against cherry-picking model architectures and latent space dimensionality, we also compare the results of the VAE-based reduction to other non-neural net methods. See Appendix C for further details.

Additionally, the sampling and backmapping of latent space points to parameter space can occur through several different methods. In what follows, we choose a particularly simple implementation of the backmapping; when sampling a new latent space goal, we look to the nearest previously sampled latent space point, and we identify its associated parameter space point. We can then make a random step from this nearest-neighbor parameter space point. In this way, we make a guess at what points in parameter space are likely to produced a dynamical behavior with our targeted latent space goal. Our choice for latent space sampling is similarly simple; we uniformly sample the bounding hypercube of the current set of collected latent space points. For further details, see Appendix B, and refer to the subsection Limitations and Extensions for a discussion of other latent space and backmapping methods.

### Relation to other methods

There is a long history of methods to explore and optimize in high-dimensional design spaces with known order parameters. These include evolutionary algorithms [10] and Bayesian optimization [12–15], which define auxiliary functions based on desired structural properties in order to direct parameter sampling. Excitingly, active learning [7,34] has been used for exploration in autonomous chemical laboratories [8,9,35], though again within a space of predetermined metrics of interest. These methods are complementary to the curiosity search described in our paper as ways for exploring the latent space of order parameters once it is constructed.

For data-driven order parameter construction in physical systems, there has been an explosion of work within the past five years. Beginning with canonical lattice models [17,36,37] and more recently in systems hosting topological [38], MBL [39], complex crystalline [19], or activity-driven phases [23, 24], deep-learning-based dimensionality reduction has been employed to extract order parameters from a diverse set of system microstates. In the straightforward approach to order-parameter construction, a data set of sufficient richness is required for training.

Our approach applies iterative algorithms originally developed in the field of intrinsically motivated robotics [27,40], which have been used more recently in the context of cellular automata [29] and gene regulatory networks [30].

## III. BENCHMARK RESULTS IN KNOWN SYSTEMS

**FIG. 2.**
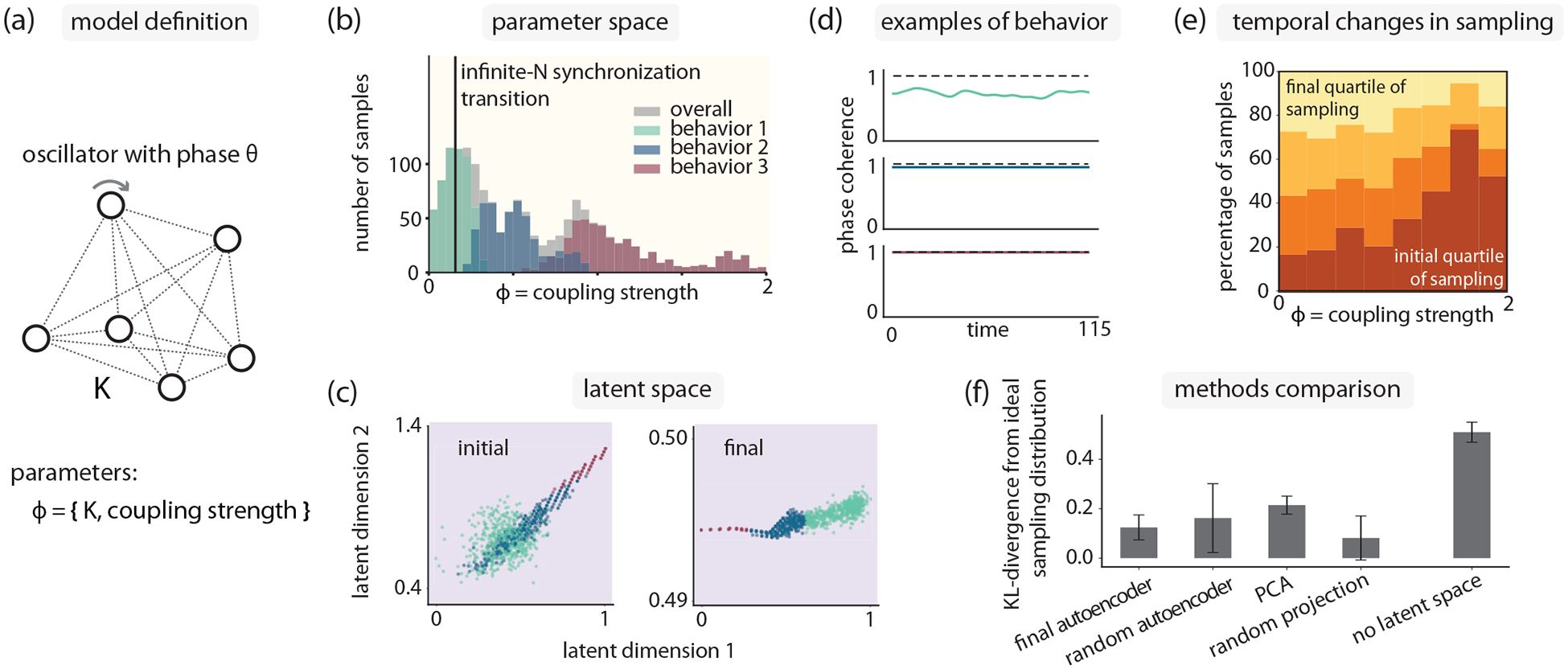
Curiosity search efficiently reveals all known phases and order parameters for the uniformly connected Kuramoto model. (a) In the canonical Kuramoto model, N oscillators are coupled to favor alignment (coupling strength K). Number of oscillators N=33. (b) Compared to a random search of K space, our algorithm samples a less common desynchronized state at small K more frequently. A vertical line indicates phase boundary $K_{c}$ computed for N→∞. Behaviors and the associated colors are computed from latent space. (c) Autoencoder latent space at the start and end of curiosity search; the final latent space identifies a one-dimensional structure for oscillator behavior, indicating one useful order parameter. Clustering in latent space helps to parse collective behaviors, corresponding to distinct regions of parameter space. (d) Phase coherence examples from dynamical states identified through latent space clustering. Examples are chosen by identifying the samples closest to the cluster median in latent space. (e) Curiosity search increasingly focuses on sampling lower K as training proceeds. (f) Curiosity search works with other dimensionality reduction methods, consistently generating better parameter sampling than random sampling (no latent space). Error bars indicate variance over 10 replicates.

As a first benchmark for performance of a curiosity-driven search in a simple, well-characterized setting, we turn to the original formulation of the Kuramoto model [31]:

(1) $${\dot{\theta }}_{i}=\omega_{i}+\frac{K}{N}\sum_{j=1}^{N}\text{sin}\left(\theta_{i}-\theta_{j}\right),$$

where the $\omega_{i}$ are drawn independently from a distribution 𝒩(0,0.1), and the coupling strength K>0 is the one tunable parameter [Fig. 2(a)]. We set N=33 for our simulations.

In the limit of infinite N, this model is characterized by a critical coupling strength [32], which is $K_{c}=0.16$ for our parameters. For $K<K_{c}$, the oscillators move independently of each other, creating a desynchronized behavior. For $K>K_{c}$, the oscillators begin to synchronize and move together with the same phase.

Let us pretend that we are approaching this system without prior knowledge about the behaviors that can arise, and where these transitions occur. In other words, the only information we have about the system is that there is one parameter that we can manipulate, which is K. One way to approach exploration of this system would be to randomly sample values of K at values of O(1), and observe the behavior at these sampled values. With this approach, only a small fraction of the observed behaviors would be desynchronized, since $K_{c}$ is O(0.1).

As a test case that should be easy, we perform curiosity search in the one-dimensional parameter space of coupling strength. We find that, in the final ensemble of collected parameters, samples are drawn with frequencies weighted towards couplings of O(0.1), where we expect the infinite-N synchronization transition to occur [Fig. 2(b)]. We can interpret this weighted sampling as the curiosity search having learned to distinguish the synchronized and desynchronized phases. The latent space also provides evidence for learning of the Kuramoto model order parameter, as the final latent space is a 1D manifold with the same ordering as the parameter space. Hierarchical agglomerative clustering, as a post-data-collection step, readily reveals this ordering by showing how contiguous regions of latent space are mapped to parameter space [Fig. 2(c)].

Individual examples of dynamical behaviors from different regions of latent space provide additional support for the idea that distance along the latent space manifold has physical significance [Fig. 2(d)]. Plotting the traditional Kuramoto phase coherence $\left|\frac{1}{N}\sum_{j=1}^{N}e^{i\theta_{j}}\right|$, we see that desynchronized (behavior 1) and synchronized (behavior 3) patterns are located on opposite ends of latent space, with partially synchronized (behavior 2) patterns in between. These examples are representative of each cluster, and they are chosen by identifying the parameters that generate the samples closest to the cluster median in latent space. Finally, we see that sampling bias towards the desynchronized region increases as sampling progresses, indicating that the curiosity search is changing its latent space over time to better reflect the relevant behaviors [Fig. 2(e)].

To test whether other algorithms could have performed the same task, we considered multiple variants of the dimensionality reduction technique: PCA, a random autoencoder that was never trained, and a random linear projection (see Appendix C for further details). As we have access to a prior understanding of the dynamical behaviors present in the model, we can compare the distribution of sampling postcollection to an ideal distribution that samples the known behaviors equally [Fig. 2(f)]. All latent space-based searches consistently outperformed random sampling of parameter space. We note the surprising result that random projection outperformed even iteratively trained methods, indicating there was enough structure present in the raw system output such that a random low-dimensional projection was able to separate the various accessible behaviors.

While the uniformly connected Kuramoto model is an ideal testing ground, the range of dynamical behaviors it can produce is fairly simple. We extend our approach to a Kuramoto model variant whose phase diagram has been equally well-characterized, but is capable of producing a wider range of behaviors, including chimera states.

Specifically, we investigate a two-population Kuramoto model with a coupling $K_{11}=K_{22}=\mu$ between all oscillators within the same population, and a coupling $K_{12}=K_{21}=v$ between all oscillators in different populations. Subscripts indicate the oscillator population index. We allow a phase offset α to the coupling between any two oscillators, and we write the model as

(2) $$\dot{\theta_{i}^{\sigma }}=\omega +\sum_{\sigma^{\prime }=1}^{2}\frac{K_{\sigma \sigma^{\prime }}}{N_{\sigma^{\prime }}}\sum_{j=1}^{N_{\sigma^{\prime }}}\text{sin}\left(\theta_{j}^{\sigma^{\prime }}-\theta_{i}^{\sigma }-\alpha \right).$$

**FIG. 3.**
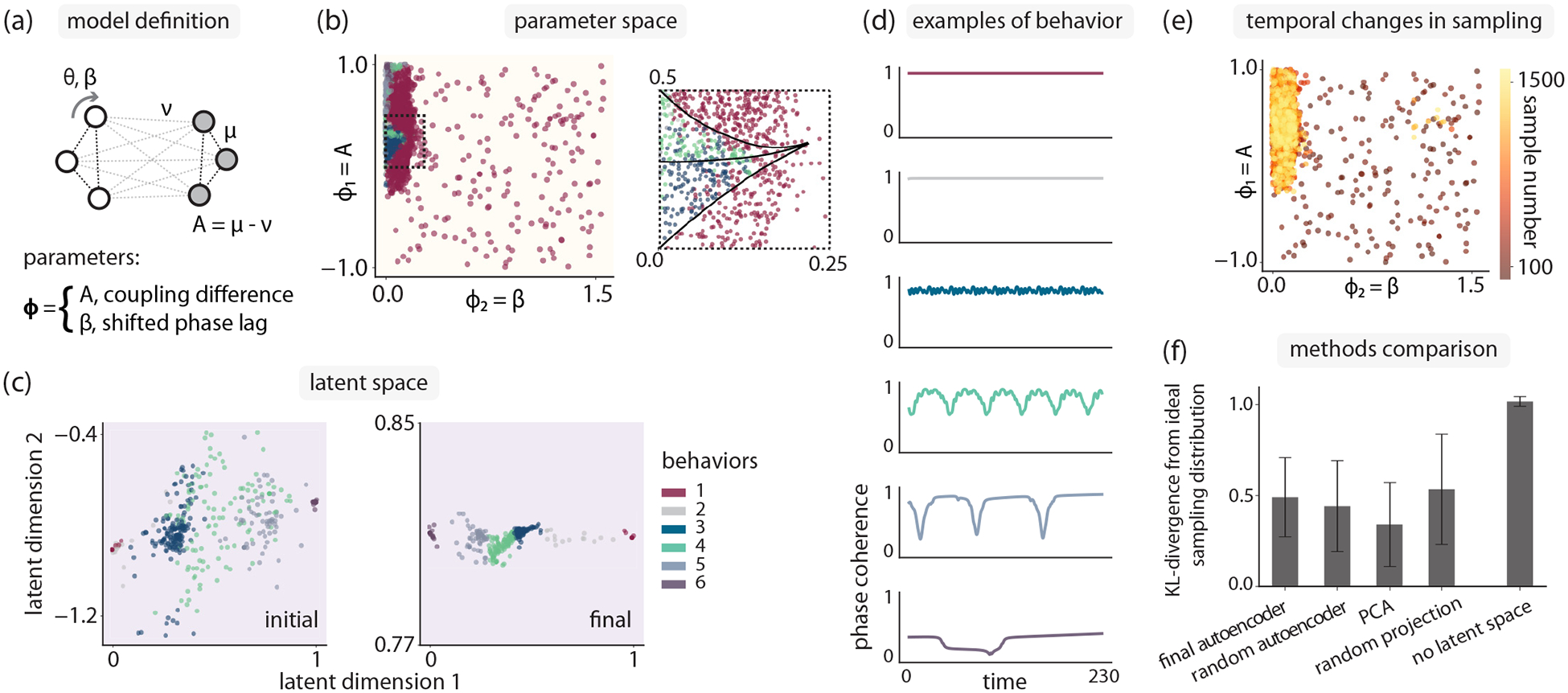
Curiosity search efficiently reveals the full phase diagram for a two-population Kuramoto model. (a) Kuramoto model with two populations of oscillators considered by Ref. [41], with intrapopulation coupling μ, interpopulation coupling ν, and shifted phase offset β, with number of oscillators N=32. (b) Curiosity search focuses sampling on the small region where rare chimera behaviors occur. Inset: Clustering in latent space reveals that this region has the structure of the chimera stability diagram identified by Ref. [41] (interior dashed lines). (c) Latent space at the start and end of curiosity search. (d) Phase coherence examples from each of the states identified through latent space clustering. Examples are chosen by identifying the samples closest to the cluster median in latent space. (e) As training proceeds, curiosity search increasingly focuses on the parameter space region where desynchronized states are found. (f) Curiosity search works with other dimensionality reduction methods, consistently generating better parameter sampling than random sampling (no latent space). Error bars indicate variance over 10 replicates.

This model was introduced by Ref. [41], where the parameter space was given by the variables $\beta =\frac{\pi }{2}-\alpha$ and A=μ−v [Fig. 3(a)], with μ+v=1. Here, we investigate the case ω=0 and total N=32, with equal population sizes. We term this model the “chimera” model, as it hosts chimera states, where two identical populations of oscillators exist with one population synchronized and the other desynchronized [42–45].

Employing curiosity search in this two-dimensional parameter space results in a distribution of samples that is concentrated on a narrow strip of the parameter space, roughly in the area with A>0 and θ<0.25 [Fig. 3(b)]. This is the region of parameter space that is known to support the emergence of chimeras. In fact, the latent space order parameter trained through our curiosity sampling procedure is able to distinguish between the two types of chimeras originally identified by Ref. [41] [Figs. 3(b) (inset) and 3(c)].

Visualizations of the dynamical behaviors provide additional evidence that automated curiosity sampling is capturing a wide variety of behaviors in the chimera model [Fig. 3(d)], and the temporal changes in sampling indicate that the parameter regions that contain the richest dynamical behaviors are preferentially sampled as the latent space is trained [Fig. 3(e)]. Behaviors 3 and 4 correspond to the breathing and stable chimeras identified by Ref. [41], respectively. Examples for each cluster are chosen by identifying samples closest to the corresponding cluster median in latent space.

As we have access to a prior understanding of some of the dynamical behaviors present in the model, we can compare the distribution of sampling postcollection to an estimated ideal distribution that samples the known behaviors equally [Fig. 3(f)]. All latent space-based searches consistently outperformed random sampling of parameter space.

## IV. RESULTS IN NEW SYSTEMS

**FIG. 4.**
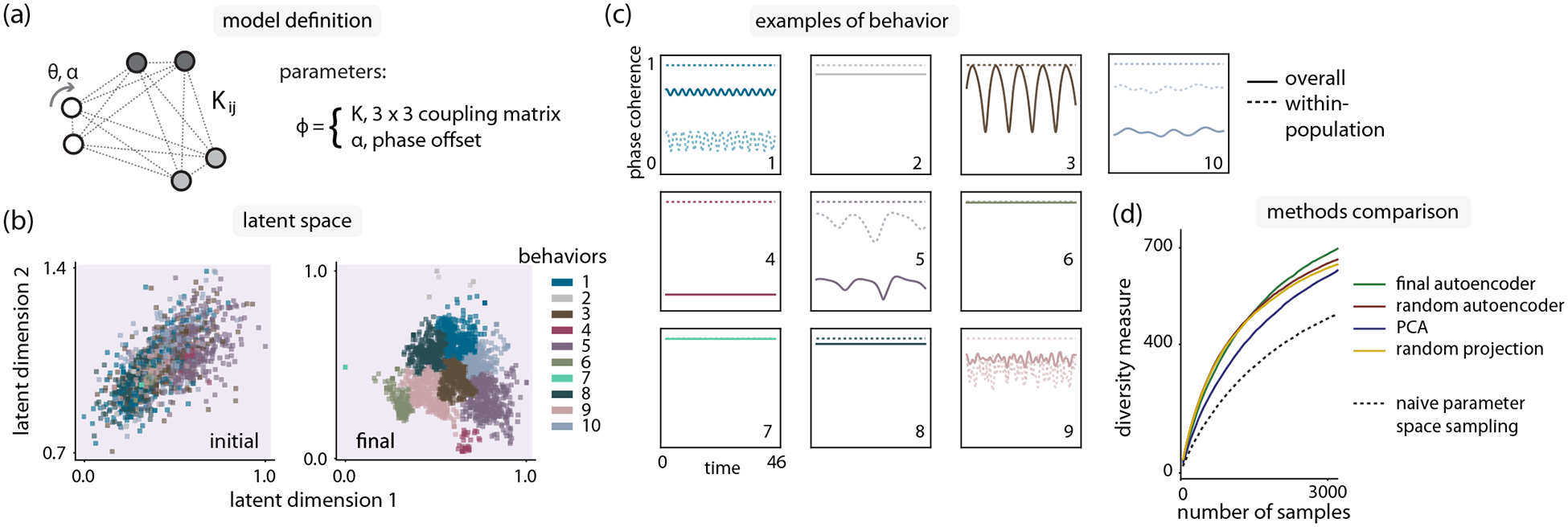
Curiosity search in a three-population Kuramoto model reveals a previously unknown phase. (a) A three-population Kuramoto model with nine positive couplings $K_{ij}$ and one phase offset α. Number of oscillators N=30. (b) Latent space at the start and end of curiosity search. (c) Phase coherence examples from each of the states identified through latent space clustering. Solid lines represent overall phase coherence, dashed lines are phase coherence of individual populations. Examples are chosen by identifying the samples closest to the cluster median in latent space. (d) All dimensionality reduction variants of the curiosity search algorithm generate more diversity than random sampling (black dashed line). Diversity is computed as the volume of sampled regions in latent space; see Appendix E2 for further details on diversity measure. Each line is computed from 10 replicates.

Having investigated the utility of automated curiosity sampling in a nontrivial but still thoroughly explored model, we now turn to unexplored models. We define a 10-dimensional variant of the chimera model, with three populations and a global phase offset [Fig. 4(a)]:

(3) $$\dot{\theta_{i}^{\sigma }}=\omega +\sum_{\sigma^{\prime }=1}^{3}\frac{K_{\sigma \sigma^{\prime }}}{N_{\sigma^{\prime }}}\sum_{j=1}^{N_{\sigma^{\prime }}}\text{sin}\left(\theta_{j}^{\sigma^{\prime }}-\theta_{i}^{\sigma }-\alpha \right),$$

with ω=0 and total N=30 divided equally among individual populations. The coupling matrix between the populations is not restricted to be symmetric, though we require all matrix elements to be positive.

To explore the four-dimensional latent space constructed through the curiosity search, we select the two dimensions in latent space that contribute the most to the largest two principal components of the trained latent space, and we project our data on these order-parameter axes [Fig. 4(b)].

To understand the behavior regimes in this latent space, we can visualize cluster-median representatives of each group for qualitative analysis. We examine both the overall phase coherence as well as the phase coherence of each individual population [Fig. 4(c)]. We find a variety of behaviors, most of which can be interpreted in light of previous behaviors uncovered in Kuramoto models—fully synchronized [31] (behaviors 6, 7), chimera [41] (behaviors 1, 9), chiral [46] (behaviors 2, 8), antialigned [46] (behavior 4), and combination chiral + chimera phases (behaviors 5, 10).

Finally, to conclude our automated analysis of the three-population Kuramoto model, we quantitatively confirm the relative diversity of samples compared to a random sampling baseline. In contrast to the uniformly connected and chimera models, we lacked any prior knowledge of the phase behavior in parameter space. We therefore adopted a model-agnostic measure of diversity corresponding to the total volume of trained autoencoder latent space occupied by another sampling distribution [Fig. 4(d)]. See Appendix E2 for further details.

In our exploration of the three-population Kuramoto model, we identified a particular set of parameters that led to an unexpected behavior [Fig. 4(c), behavior 3], where the phase coherence of each individual oscillator population was saturated, but the overall phase coherence displayed periodic variability. We were particularly interested in understanding this behavior, as it did not neatly fit into any categories that we had previously encountered, resembling a chiral phase identified in Fruchart *et al*. [46], but with periodic breathing.

**FIG. 5.**
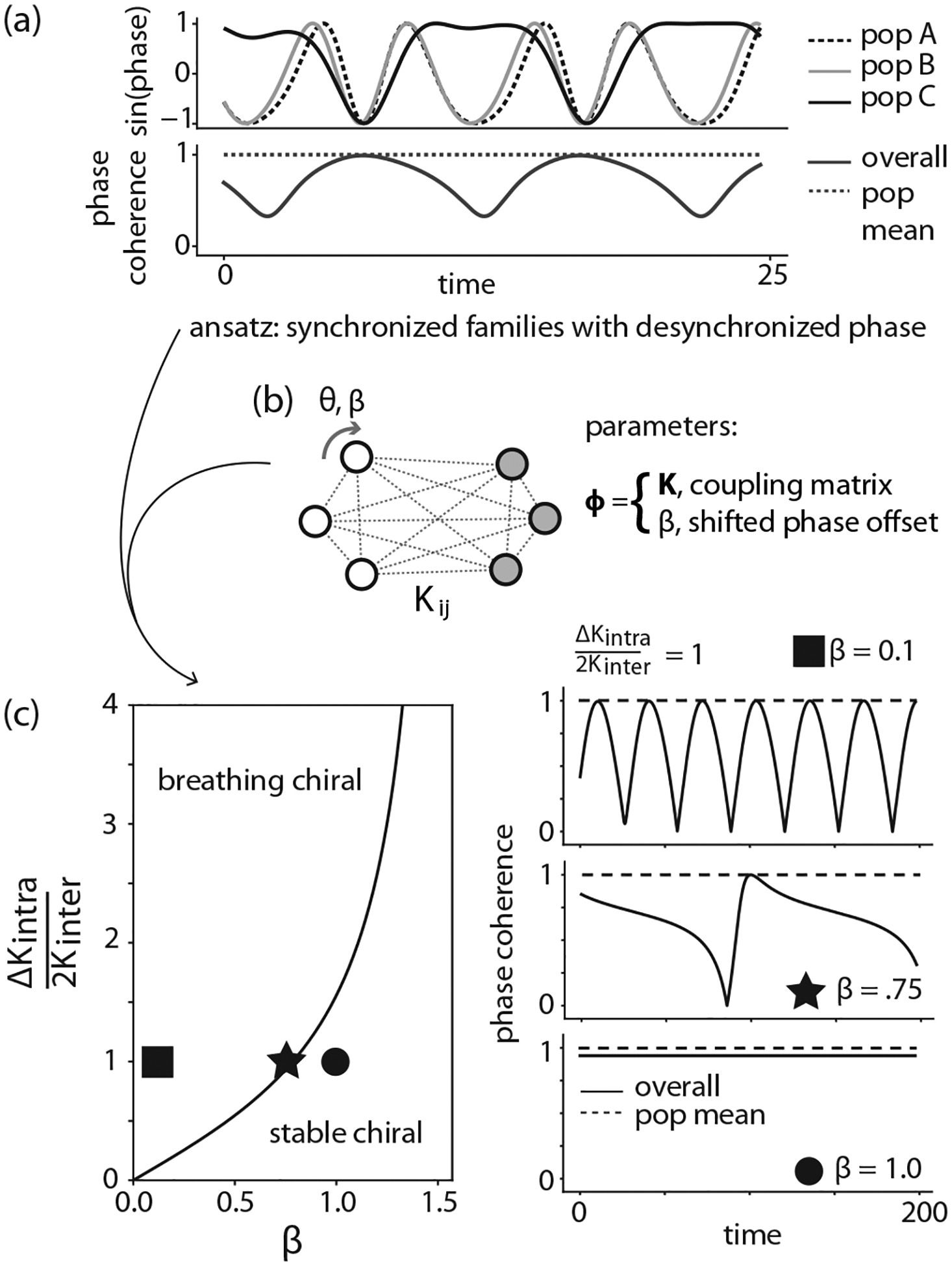
Automated curiosity search in a three-population Kuramoto model yields transferable insights for other models. (a) One behavior found in the Fig. 4(c) curiosity search shows complete synchronization within populations, but with one population desynchronized from the other two: a “chiral breather” state. The dashed population mean indicates the average of the three phase coherence curves for the individual populations, with number of oscillators N=30. (b) Qualitative behavior can be used as an ansatz for solving a simpler two-population Kuramoto model (infinite-N limit), with coupling matrix $K_{ij}$ and shifted phase offset β. (c) Solution to the two-population model with new ansatz reveals the phase diagram of breathing and stable chiral states as a function of population couplings and phase offset (left). Dynamics of overall and population mean phase coherence at specific points in phase diagram, with number of oscillators N=32 (right).

We took a closer look at these “chiral breather” dynamics, and we found that the behavior came as a result of two populations completely synchronizing with each other, while a third population internally synchronized but moved at a different period relative to the other populations [Fig. 5(a)].

To understand the chiral breather, we looked for solutions with internally synchronized populations with an externally desynchronized phase in a simpler system. We chose to investigate a two-population version of the three-population model [Fig. 5(b)], which is identical to Eq. (2), without the inter- and intrapopulation coupling symmetry assumptions:

(4) $$\dot{\theta_{i}^{\sigma }}=\omega +\sum_{\sigma^{\prime }=1}^{2}\frac{K_{\sigma \sigma^{\prime }}}{N_{\sigma^{\prime }}}\sum_{j=1}^{N_{\sigma^{\prime }}}\text{sin}\left(\theta_{j}^{\sigma^{\prime }}-\theta_{i}^{\sigma }-\alpha \right).$$

Following the procedure outlined in Ref. [41], we derive a set of coupled differential equations for the phase difference and coherence of the two oscillator populations in the limit of infinite population size $N_{\sigma }\to \infty$ for σ=1,2. In this limit, the governing continuity equation becomes

(5) $$\frac{\partial f^{\sigma }}{\partial t}+\frac{\partial }{\partial \theta }\left(f^{\sigma }v^{\sigma }\right)=0,$$

where $f^{\sigma }(\theta ,t)$ is the per-population oscillator density and $v^{\sigma }(\theta ,t)$ is the oscillator density velocity:

(6) $$v^{\sigma }\left(\theta ,t\right)=\omega +\sum_{\sigma^{\prime }=1}^{2}K_{\sigma \sigma^{\prime }}\int \text{sin}\left(\theta^{\prime }-\theta -\alpha \right)f^{\sigma^{\prime }}\left(\theta^{\prime },t\right)d\theta^{\prime }.$$

Equations (5) and (6) have solutions following the remarkable Ott-Antonsen ansatz [47]:

(7) $$f^{\sigma }\left(\theta ,t\right)=\frac{1}{2\pi }\left[1+\sum_{n=1}^{\infty }\left\{{\left[a_{\sigma }\left(t\right)e^{i\theta }\right]}^{n}+\text{c.c.}\right\}\right],$$

where c.c. denotes the complex conjugate of the nth term, and $a_{\sigma }(t)$ are amplitudes that define the full time-dependent solution for the oscillator population densities.

Returning to Eqs. (5) and (6) with this solution form, we arrive at the equations for the population amplitudes as in Eq. (9) in Ref. [41]:

(8) $$0={\dot{a}}_{1}+\frac{1}{2}a_{1}^{2}\left(K_{11}a_{1}^{*}+K_{12}a_{2}^{*}\right)e^{-i\alpha }-\frac{1}{2}\left(K_{11}a_{1}^{*}+K_{12}a_{2}^{*}\right)e^{i\alpha },$$

with the equation for ${\dot{a}}_{2}$ being identical under the interchange of subscripts 1 and 2.

Note that, since Eq. (7) is a rewriting of a Poisson kernel, the amplitudes $a_{\sigma }$ provide a key physical interpretation of the time evolution of the oscillator densities. If we write amplitudes $a_{\sigma }=\rho_{\sigma }e^{-i\phi_{\sigma }}$, the $\rho_{\sigma }(t)$ reflect the phase coherence of the population oscillator densities, while the $\phi_{\sigma }(t)$ represent the center of the oscillator densities. Inspired by their observations, Ref. [41] looked for amplitude solutions with a synchronized population ($\rho_{1}=1$) and a desynchronized population ($\rho_{1}<1$).

Our observations suggested that we should employ a different amplitude ansatz in order to capture the behavior exhibited in Fig. 5(a), with internally synchronized populations and a desynchronized phase. We look for solutions where $\rho_{\sigma }=1$ for both σ. In this case, for $a_{\sigma }=e^{-i\phi_{\sigma }}$, Eq. (8) reduces to

(9) $$0={\dot{\phi }}_{1}+K_{11}\;\text{sin}\;\alpha +K_{12}\;\text{sin}\left(\alpha +\phi_{1}-\phi_{2}\right),$$

with the associated equation for index 2 simply involving the exchange of subscripts for 1 and 2. We can define $\psi =\phi_{1}-\phi_{2}$, in which case we have one equation:

(10) $$\dot{\psi }=-\left[\left(K_{11}-K_{22}\right)\;\text{sin}\;\alpha +K_{12}\;\text{sin}(\alpha +\psi )-K_{21}\;\text{sin}(\alpha -\psi )\right].$$

Integrating yields

(11) $$\psi (t)=2\;{\text{tan}}^{-1}\left[\frac{D\;\text{tan}\left(-\frac{Dt}{2\sqrt{2}}+c_{0}\right)-A}{B}\right],\;A=\left(K_{12}-K_{21}\right)\text{cos}\;\alpha ,\;B=\sqrt{2}\;\text{sin}\;\alpha \left[\left(K_{11}-K_{22}\right)-\left(K_{12}-K_{21}\right)\right]\;D=\left\{{\left(K_{11}-K_{22}\right)}^{2}-2\left(K_{12}^{2}+K_{21}^{2}\right)\right.{\left.-\left[{\left(K_{11}-K_{22}\right)}^{2}+4K_{12}K_{21}\right]\text{cos}\;2\alpha \right\}}^{\frac{1}{2}},$$

where $c_{0}$ is a constant of integration.

We note that there are two behaviors embedded in this solution, depending on Im(D). When D is real, ψ continues to change over time as t→∞, indicating a chiral breather. If D is imaginary, then because of the relation between tan and tanh for imaginary arguments, ψ goes to a constant in the long-time limit, indicating a stable chiral phase. In the case where $K_{12}=K_{21}=K_{\text{inter}}$ and we define $\Delta K_{\text{intra}}={\left(K_{11}-K_{22}\right)}^{2}$, the boundary between these two behaviors simplifies to

(12) $$\Delta K_{\text{intra}}^{2}=4K_{\text{inter}}^{2}{\text{tan}}^{2}\;\beta ,$$

where $\beta =\frac{\pi }{2}-\alpha$ is the shifted phase offset.

Therefore, using our ansatz inspired from our data-driven exploration in Fig. 4, we were able to compute the steady-state behavior of the oscillators as a function of the model parameters [Fig. 5(c) (left)]. Indeed, when we simulate specific parameters with N=32 oscillators [Fig. 5(c) (right)], we find this transition from chiral breather to stable chiral behavior, as predicted from the infinite-N analysis.

## V. DISCUSSION

### A. Limitations and extensions

While our method is successful in identifying novel phases and order parameters with minimal human effort, there are limitations on the effectiveness of our curiosity search as currently implemented. Many of these limitations can be traced to the geometry of the parameter space-to-behavior space map.

One set of issues comes from the strength of gradients in behavior as a function of design parameters. If the behavior is constant in a region of parameter space, then our choice to randomly sample locally to previously explored parameter values can result in search dynamics that are equivalent to diffusion in that region of parameter space. This local diffusion can result in a heavy dependence upon the behaviors initially sampled to seed the curiosity search. The problem becomes more acute as the dimension of parameter space increases.

This limitation suggests that finite-size systems, away from thermodynamic limits with sharp transitions in behaviors, may be more amenable to methods of curiosity search that operate in the space of behaviors. There may be hints of one type of behavior hidden in examples of another behavior, and hence our method can follow changes in behavior, rather than relying solely on diffusion to randomly find a phase boundary. However, in the case of both diffusive and gradient-following dynamics, we expect that whenever a new behavior is discovered, the curiosity search algorithm will sample it with elevated frequency.

A key part of the curiosity search framework is the backmapping from behavior space to parameter space. Our nearest-known-neighbor choice was particularly simple, and as discussed, potentially introduces a decrease in exploration efficiency and an increased dependence on initial conditions when sampling in higher dimensions. One possibility for decreasing the reliance on previously sampled parameters is to translate geometrical information in behavior space back into parameter space. For example, if a target behavior sampled in behavior space lies between two points, we might sample between the two corresponding points in parameter space. Another possibility would be to learn the backmapping as a supervised deep-learning problem, iteratively updating the backmapping as latent space changed.

Another key component of the curiosity search framework for which we made a simple choice was in latent space sampling policy. While our current methodology samples latent space uniformly, it might be more efficient to explicitly sample in regions of latent space which have lower sample densities, or to sample specifically on the boundaries of those latent space regions.

**FIG. 6.**
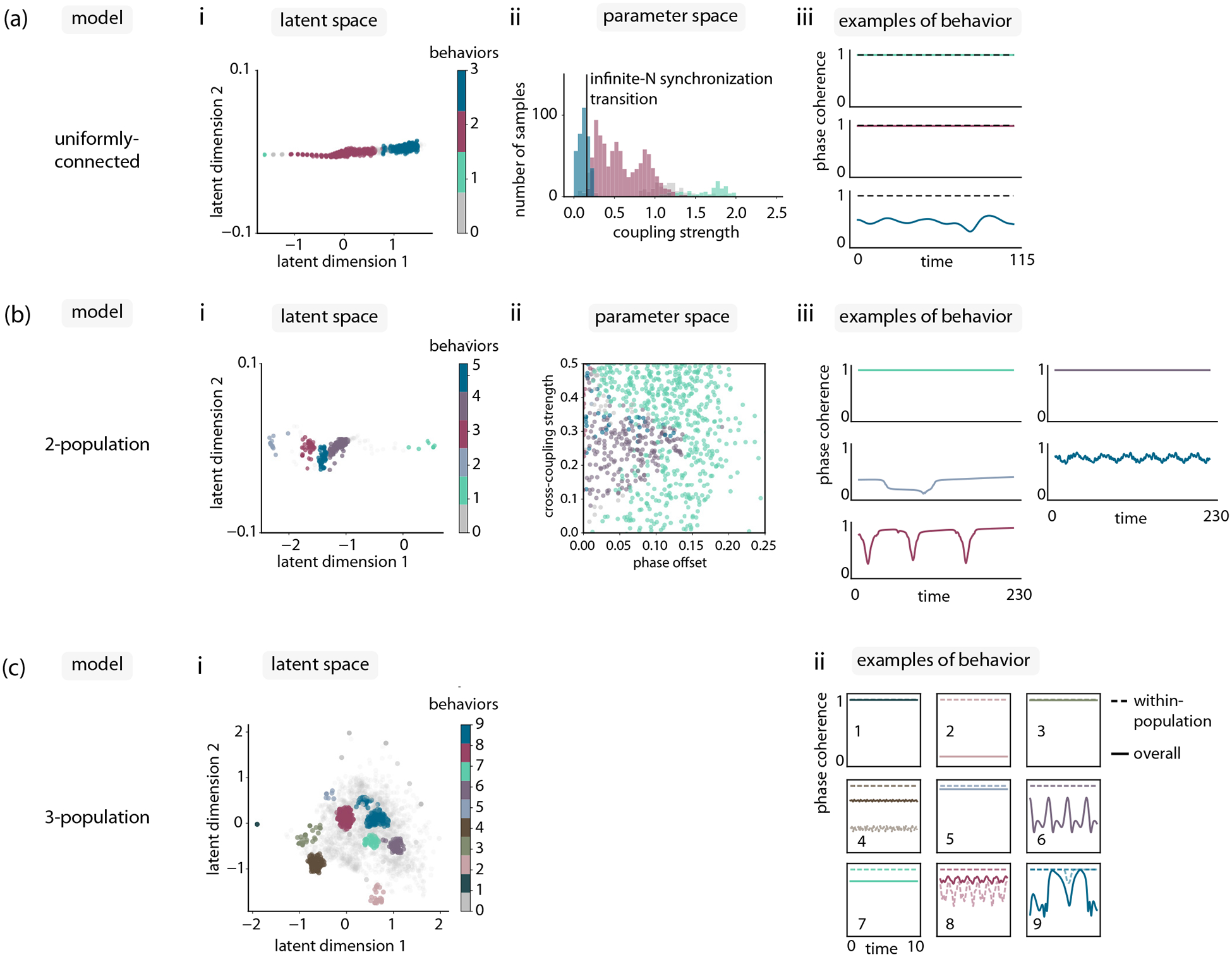
Clustering with HDBSCAN on curiosity search data reveals novel dynamical behaviors in Kuramoto model variants. In all cases, examples of behaviors are chosen to be closest to latent space cluster medians. (a) Clustering with HDBSCAN in the latent space of the uniformly connected model identifies three clusters (i), which map to regimes of low, intermediate, and high synchronization in parameter space (ii,iii). (b) Clustering with HDBSCAN in the latent space of the chimera model identifies five clusters (i) which can roughly distinguish between chimeric and fully synchronized phases (ii,iii). (c) Clustering with HDBSCAN in the latent space of the three-population model identifies nine clusters (i), which reveal similar behaviors to those identified in the main text (ii), including the chiral breather behavior (behavior 6).

Finally, we made choices in the analysis of our latent space post-data collection, in particular performing agglomerative clustering on these data. We emphasize that the clustering is a computational device to render the latent space more human-interpretable, but is not crucial for the success of the algorithm; we could equally well have simply binned latent space. However, to check to make sure that the choice of clustering algorithm does not significantly change the interpretation of the latent space and associated behaviors identified, we performed clustering with HDBSCAN [48] on all data sets generated, and we were able to *de novo* discover the same interesting behaviors as identified in postprocessing with agglomerative clustering (Fig. 6). Additionally, while agglomerative clustering requires us to specify a number of expected phases, we found that HDBSCAN automatically chose similar numbers of phases when the minimum cluster size was set to 2% of the total data-set size (Fig. 6).

In cases with only partial observations, time-delay embedding can be used to capture the full structure of the attractor. However, questions of time and resource cost of experimental iterations and the effectiveness of our method with only partial observations remain to be explored.

A final extension, which we demonstrate more fully in Appendix F, is the iterative incorporation of human-in-the-loop feedback in order to guide sampling towards more subjectively interesting behaviors.

### B. Conclusion

We have demonstrated that it is feasible to explore dynamical systems despite not knowing how to characterize the salient features of their behaviors (e.g., in terms of order parameters). This *curious* exploration enables us to learn the metrics that characterize a novel system without having a predefined target or goal [27,49]. We achieved this curious exploration by combining the complementary strengths of active learning and dimensionality reduction; dimensionality reduction enables the iterative construction of a low-dimensional latent space of behaviors, while searching in latent space improves the efficiency of data collection. While active learning and dimensionality reduction have individually been applied in the context of physical systems, this approach allows us to solve a qualitatively new challenge that has yet to be confronted in a physical domain.

While we applied curiosity search to a canonical but *in silico* model of a complex system, our algorithm can instead directly interface with a physical system by taking control of experimental knobs. This direction will allow us to discover functional behaviors that exploit unmodeled or unexpected effects in experimental systems such as nonlinearities [50] or feedback. Much like reservoir computing [51] or model-free control [52], our work here provides a systematic way of revealing behaviors that exploit complex unmodellable effects, rather than discovering them through serendipity.

Natural applications along these lines include active matter systems with spatial structure. Recent experimental advances increasingly allow for the control of activity [1,4] and particle interactions [5,6] in a space-time-dependent manner, allowing for detailed density- and orientation-dependent motility. These experimental methods have opened up complex high-dimensional spatiotemporal design spaces; since order parameters are typically not available *a priori* for these systems, the methods in this work provide exciting opportunities for revealing novel behaviors.

## Figures and Tables

**FIG. 7.**
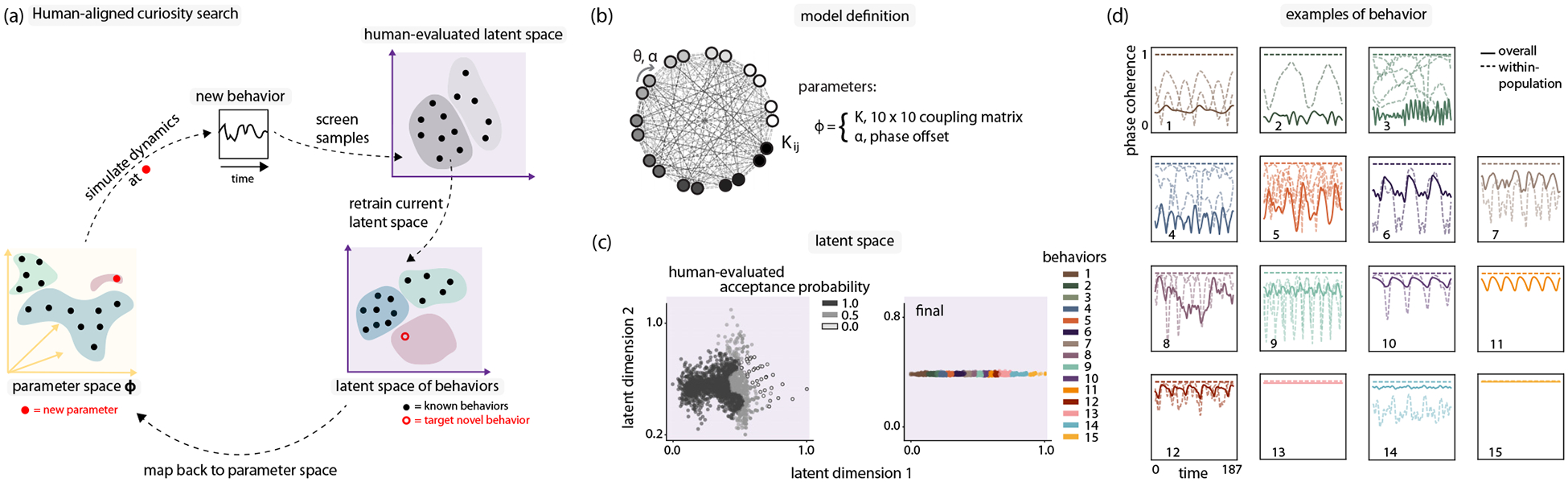
Human-aligned curiosity search discovers chaotic multipopulation behaviors in the 100-dimensional Kuramoto model. (a) Curiosity search can naturally be aligned with human intuition with a single additional step, involving freezing the latent space of an initial curiosity searcher. Behavior samples drawn for subsequent curiosity search can be screened through the frozen latent space, where samples are accepted or rejected based on probabilities assigned to different parts of latent space by a human observer. Following screening through this human-evaluated latent space, sampling proceeds as in Fig. 1, with a separate autoencoder for the new, human-aligned latent space. (b) A 10-population Kuramoto model with 100 positive couplings $K_{ij}$, and one global phase offset α. Number of oscillators N=100. (c) Left: Latent space following initial curiosity search without human alignment. Latent space clusters identified in initial search are human-evaluated for interest, and assigned acceptance probabilities. Right: Final latent space at the end of human-aligned curiosity search. (d) Phase coherence examples from each of the states identified through latent space clustering. Solid lines represent overall phase coherence, dashed lines are phase coherence of individual populations. Examples are chosen by identifying the samples closest to the cluster median in latent space.

## APPENDIX A: SIMULATION DETAILS

Our primary goal is to investigate and discover novel dynamical behaviors in variants of the Kuramoto model, all of which can be compactly written in the following form:

(A1)
$${\dot{\theta }}_{i}=\omega_{i}+\sum_{j=1}^{N}K_{ij}\;\text{sin}\left(\theta_{j}-\theta_{i}-\alpha \right),$$

where $K_{ij}$ is the matrix of couplings between oscillators, α is a global phase offset, the intrinsic frequencies $\omega_{i}$ are (potentially) drawn from a distribution, and N is the number of oscillators. In all figures, N is generally on the order of 30, and $\omega_{i}=0$ except for in the uniformly connected model considered in Fig. 2. In the uniformly connected model, $\omega_{i}$ is drawn from the distribution 𝒩(0,0.1). Additionally, in the uniformly connected model, α=0.

To investigate these dynamical systems, we need to integrate Eq. (A1) for specific $K_{ij}$ and α, for which we use the SciPy odeint function. We supply a regular time grid of domain [0, 750] with step size dt=0.05. Initial oscillator phases are drawn from a uniform distribution from [0,2π].

Traditionally, the output of these models has been investigated using a “phase coherence” order parameter, which can be defined as $\left|\frac{1}{N}\sum_{j=1}^{N}e^{i\theta_{j}}\right|$. There is also the associated complex phase $\text{arg}\left[\frac{1}{N}\sum_{j=1}^{N}e^{i\theta_{j}}\right]$, but we focus on this less in our current work.

Instead of interpreting and processing the output of our dynamical system with these traditional metrics, we allow unsupervised dimensionality reduction techniques (see Appendix C) to extract the relevant order parameters. To pass the raw output of the dynamical systems integration to the dimensionality reduction technique, we sample the last 3% of the raw output time series in seven evenly spaced intervals. At each sample, we use the NumPy arctan2 function to compute the mean oscillator phase, and then we compute the distribution of sines of oscillator angles relative to the mean. The sine values are subsequently binned in the range [−1, 1] with seven bins, and the histogram is normalized by the number of oscillators. Having done this for seven time points, we have transformed our raw dynamical output into a 7 × 7 gray-scale image, which is the input to a dimensionality reduction technique. Note that by mean-centering at each time point, and binning, we imposed invariance to the oscillation index, as well as invariance to global rotations. In the context of coupled oscillator models, these assumptions seem relatively benign, but they may not be appropriate for other systems.

## APPENDIX B: ACTIVE LEARNING DETAILS

Having described how we compute the behavior for a given set of Kuramoto model parameters, we can now turn to the active learning procedure by which we sample model parameters. Our explorations are seeded by collecting 200 (uniformly connected and chimera models) or 800 (three-population model) samples randomly throughout parameter space. Each parameter space axis has an upper and lower bound: for phase offsets, this is $\left[0,\frac{\pi }{2}\right]$; for oscillator couplings, this is [0, 1] in the chimera and three-population models, and [0, 2] in the uniformly connected model.

The parameters are then used to integrate the dynamical equation described in Appendix A. We note that, for the chimera and three-population models, initial oscillator phases are sampled once at the beginning of the active learning procedure, and subsequently fixed for the duration of the exploration. In contrast, for the uniformly connected model, the initial oscillator phases and the intrinsic frequencies are resampled with each new parameter selected. The output of these initial simulations is passed through a dimensionality reduction technique (Appendix C). If the employed technique requires training, training is also performed before the dynamical behaviors are converted to their latent space representations.

We have now initialized our active learning exploration by creating a collection of tuples containing all our relevant information (parameters, dynamic behaviors, latent space representation). Following initialization, we now select a “target” behavior in latent space that we wish to explore. While there are many possible options for performing this latent space sampling, we pick a particularly simple one; we construct the hyper-rectangle that contains all the currently sampled latent space representations, and then uniformly sample within that hyper-rectangle.

With this target behavior in hand, we now seek a point in parameter space that will ideally lead us to this target point in latent space. Again, there are many possible options for implementing this parameter point selection, and we choose a simple one. In this case, we return to our dictionary of all previously sampled parameters, and we select the parameter whose latent space representation is closest to our target. We then “mutate” this selected parameter by adding a random amount along each parameter space axis. The magnitude of this random step along each parameter space axis is bounded by 10% (uniformly connected and chimera models) or 20% (three-population) of the allowed domain length of that axis. Within these bounds, the step length is sampled uniformly. If the mutated parameter falls outside the lower or upper bound of any of the parameter axes, we resample the mutation until the mutated parameter falls within the allowed ranges.

We can continue this process, collecting more (parameter, dynamic behavior, latent space representation)-tuples. We can also iteratively train the associated dimensionality reduction techniques with this newly collected data. For the uniformly connected and chimera model explorations, we updated the dimensionality reduction technique once every 100 samples collected until we had a total of 1600 collected samples, inclusive of the initial samples. For the three-population model exploration, we updated the dimensionality reduction technique once every 400 samples collected until we collected a total of 4000 samples. When we select samples for training, we utilize 50% from the most recent samples, and 50% randomly chosen from the previous samples. All latent space values correspond to the most recent version of the dimensionality reduction technique.

## APPENDIX C: DIMENSIONALITY REDUCTION DETAILS

For each coupled oscillator model exploration, we consider four dimensionality reduction techniques in the paper: a convolutional variational autoencoder (VAE), a random VAE, PCA, and random projection. All the neural network code was run using PyTorch, and the linear models were implemented with Scikit-learn.

The convolutional VAE has a relatively simple encoder architecture: (i) a 2D convolutional layer with two to four filters, followed by ReLU activation and flattening; and then (ii) a fully connected layer into a latent space of dimension 2–4. The decoder follows analogously: (i) a fully connected layer that expands from the latent space, followed by unflattening, and ReLU activation; and then (ii) a transposed 2D convolution followed by sigmoid activation. For the chimera and uniformly connected models, we use two filters and two latent dimensions, whereas for the three-population model we use four filters and four latent dimensions. Every time the VAE is trained, it is trained on a batch size of 200 or 800 (chosen as described in Appendix B) for 2000 epochs. We train with ADAM using a learning rate of $1\times 10^{-3}$ and weight decay $1\times 10^{-5}$. Weights are initialized with PyTorch standard initialization, which in linear and convolutional layers with ReLU activation is the normalization. The random VAE is constructed in exactly the same architecture and initialization as the corresponding trained VAE, but it is never trained over the course of the active learning.

PCA is performed using the Scikit-learn PCA method, and we utilize the same number of dimensions as used for the VAEs in order to construct the PCA latent space. Random projection is performed using the Scikit-learn GaussianRandomProjection function, projected onto the same number of dimensions as used for the VAE latent spaces.

## APPENDIX D: CLUSTERING AND BEHAVIOR EXAMPLE SELECTION

To interpret latent space, we employ agglomerative clustering as implemented in the scikit-learn AgglomerativeClustering function with Ward linkage and a Euclidean metric. We choose three, six, and ten clusters for Figs. 2, 3, and 4, respectively. To gain a qualitative understanding of these clusters, we select the sample closest to the cluster median (in latent space), and then we assess the resulting dynamics for the corresponding point in parameter space. The precise values of the selected parameters are presented in supplemental Table 1 [53]. Note that in the three-population model, the listed couplings are normalized to 1 before being used as model input.

To evaluate the robustness of our clustering-related conclusions, we also perform clustering using HDBSCAN [48] on the same data set analyzed in the main figures. In contrast to agglomerative clustering, HDBSCAN does not require the number of desired clusters as a hyperparameter. Hence as a first check on the consistency of our results, we checked whether our chosen agglomerative cluster numbers could be reproduced with reasonable values of the HDBSCAN hyperparameter min_cluster_size, which sets the minimum allowable cluster size. For the uniformly connected model, we were most interested in coarse features, so we set a minimum cluster size of 100 (out of 1600 samples) to select three clusters [Fig. 6(a), i]. For the chimera model, we were more interested in fine-grained distinctions, so we set a minimum cluster size of 20 (out of 1600 samples) to select five clusters [Fig. 6(b), i]. For the three-population model, we were again interested in fine-grained distinctions, so we set a minimum cluster size of 80 (out of 4000 samples) to select nine clusters [Fig. 6(c), i].

As a second check on the robustness of our results, we asked whether we could identify the same interesting phases we found using agglomerative clustering in an HDBSCAN-derived clustering. Note that HDBSCAN identifies a category of points as noise, which in all panels we color as gray, and we label as behavior 0. For the uniformly connected model, we again recovered the low (behavior 3), intermediate (behavior 2), and fully synchronized (behavior 1) regimes [Fig. 6(a), iii]. For the chimera model, we were able to distinguish chimeric (behaviors 4 and 5) from fully synchronized regimes (behavior 1) [Fig. 6(b), ii]. There is also some distinction between breathing and stable chimeras, though the splitting is less clean than in the agglomerative clustering case. This suggests that the latent space is capable of distinguishing between the two chimera variants, but that this particular clustering is too coarse to cleanly find the dividing line. Finally, we again discover a similar range of behaviors in the three-population model to that under the agglomerative clustering analysis: fully synchronized (behaviors 1, 3), chimera (behaviors 4, 8), chiral (behaviors 7, 5), antialigned (behavior 2), chiral breathers (behavior 6), as well as behaviors with some combination of chimeric and chiral characteristics (behavior 9) [Fig. 6(c), iii].

## APPENDIX E: ALGORITHM PERFORMANCE METRICS

### 1. Ideal sampling comparison incorporating prior model knowledge

To assess the performance of the various parameter exploration schemes outlined in the main text, we want to quantify the quality of the sampling distributions they generate in parameter space. In particular, an ideal benchmarking measure would compare curiosity searches against a known, desired, sampling distribution.

In the uniformly connected Kuramoto model, we have prior knowledge about the various phases we expect to see. In the infinite-N limit, we know that there are two well-defined phases: a fully incoherent phase in which the Kuramoto order parameter r=0, and above a critical coupling $K_{c}$ a synchronized parameter regime in which r>0. With this prior knowledge, we would ideally like our sampling to be evenly distributed, with half the samples coming from above $K_{c}$ and half below. However, our simulations are performed with finite N, and hence we should not expect such cleanly delineated phases.

Instead, we define an ideal sampling by the computation of r in our simulations as a function of K. We select three regimes: one phase for which r<0.3, with the corresponding parameter values K<0.13; one for which r>0.95, and hence K>0.34; and the phase intermediate to those two. Since we have chosen to sample latent space uniformly, we posit that the ideal sampling distribution should be evenly distributed between these three regimes in parameter space.

To quantitatively make the comparison between our sampled distributions and the ideal distribution, we use the scipy.special.kl_div function to compute the KL divergence $D_{\text{KL}}$ (sampled|ideal) between the two distributions. This is the number we use as our performance metric for a particular sampling distribution.

We also have ground truth knowledge in the case of the chimera model, allowing us to compare the quality of sampling distributions analogously. We identify three phases as stable chimeras, breathing chimeras, and synchronized phases outside of the previous two regions. These phases, and their boundaries in parameter space, were identified by Abrams *et al*. [41]; while other phases might in principle exist, we do not incorporate this possibility into the analysis.

Based on the work of Abrams *et al*. [41], we can estimate these boundaries using the shapely python package to define points that lie within the triangle [(0, 0), (0, 0.2679), (0.2239, 0.3372)] to be stable chimeras; points that lie within the triangle [(0, 0.2679), (0.2239, 0.3372), (0, 0.5)] to be breathing chimeras; and points outside these triangles to be synchronized phases. Given this procedure of computing phases in parameter space, we follow the same procedure as we did for the uniformly connected Kuramoto model; we assume ideal sampling is even across the three phases, and then we compute the KL divergence between each sampling distribution and ideal sampling.

### 2. Model-agnostic diversity and entropy measures

Running the curiosity algorithm with different dimensionality reduction techniques (see Appendix C) generates different distributions of samples. We would like to compare the performance of each technique in terms of generating a more diverse collection of samples, relative to a random parameter sampling baseline.

To compare sampling distributions, we must construct a measure of diversity. For the uniformly connected and chimera models, we have prior knowledge of how many phases exist, so we can simply characterize how well the different techniques sample these known phases. However, for the three-population model, we do not have this prior knowledge. We want to construct a diversity measure that does not depend on prior knowledge.

One way to do this is to construct a measure that captures the diversity of sampling in latent space, as latent space is a representation of the system behaviors. However, each dimensionality reduction technique constructs a different latent space. We make the assumption that the trained autoencoder latent space is the most relevant latent space, and so to create comparable representations, we run each distribution of collected dynamical behaviors through the same trained autoencoder. For this diversity measure analysis, we take only the two dimensions that contribute the most to the first two principal components of the latent space data. We then normalize the autoencoder latent-space based on the full collection of latent-space representations from all distributions, so that the dimension with the largest span lies from 0 to 1; the other dimension is scaled by the same factor.

Finally, to calculate a measure of diversity for a distribution of samples, we divide each dimension of the normalized latent space into 50 bins. This divides the latent space into squares, the size and number of which are determined by the bin number. Each latent space value fits into one of these cubes. We subsequently define our measure of diversity for our sample distribution as the number of unique cubes occupied by all samples. Note that the number of samples that lie in each cube is not considered, only the number of unique cubes filled. This essentially amounts to the diversity measure being the area each data set occupies in the latent space of Fig. 4(b) (right). To generate Fig. 4(d), we repeat this calculation over 10 replicates; the averages are shown. Initial random sampling is removed for diversity measure calculation.

#### 3. Temporal sampling of the uniformly connected model

To construct a representation of how the uniformly connected model sampled over time with a periodically retrained autoencoder, we divided the parameter space (coupling strength) into eight bins, and then normalized each bin individually from 0% to 100%. Since the samples were saved in the order in which they were collected, dividing the array of samples into quartiles is equivalent to dividing it into four sequential temporal bins. We plotted what percentage of the samples in each parameter space bin lay in each quartile. This method of visualization shows where the algorithm preferentially sampled over time.

## APPENDIX F: HUMAN-ALIGNED CURIOSITY SEARCH

To demonstrate the flexibility and capacity of our curiosity search framework, we present an algorithmic extension that naturally incorporates human insight [Fig. 7(a)]. We employ this human-aligned approach to explore a 100-dimensional model.

In particular, we define a 10-population Kuramoto model with global phase offset α [Fig. 7(b)]:

(F1)
$$\dot{\theta_{i}^{\sigma }}=\omega +\sum_{\sigma^{\prime }=1}^{10}\frac{K_{\sigma \sigma^{\prime }}}{N_{\sigma^{\prime }}}\sum_{j=1}^{N_{\sigma^{\prime }}}\text{sin}\left(\theta_{j}^{\sigma^{\prime }}-\theta_{i}^{\sigma }-\alpha \right),$$

with ω=0, and total N=100 divided equally among 10 individual populations. We restrict all elements of $K_{\sigma \sigma^{\prime }}$ to be positive, and further require them to sum to 1. This model is therefore 100-dimensional. Initial exploration of the 10-population model through our non-human-aligned procedure identified several interesting behaviors, but many of the nontrivial dynamics were confined to a single population. Having previously discovered such behaviors in the three-population Kuramoto model, we no longer considered these behaviors to be novel, and we decided to prioritize the discovery of behaviors involving multiple populations.

To focus sampling on multipopulation behaviors, we introduce the concept of human evaluation of latent spaces, inspired by the HOLMES algorithm [54]. Our human-aligned curiosity search relies on the construction of an initial latent space following the procedure outlined in Fig. 1. We first performed a naive curiosity search with an autoencoder with eight latent dimensions and eight filters in the initial convolutional layer. Input to the autoencoder was constructed as before, but with 13 bins for computing oscillator phase space density as opposed to the original 7. These densities were computed at 13 time points as opposed to 7. In this initial search, we collected a total of 4000 samples, and we updated the dimensionality reduction technique once every 400 samples.

We subsequently freeze the initial latent space and assign acceptance probabilities to each cluster, based on a human evaluation of the cluster’s interest. We now perform curiosity-driven search in a new latent space, but with sampled behaviors filtered through the initial latent space; samples are rejected or accepted based on the accepted probability of the cluster they are best associated with in the initial latent space [Fig. 7(c), right].

In this particular case, we scored clusters based on the presence of behaviors involving the simultaneous presence of nontrivial dynamics in multiple oscillator populations. Therefore, the new latent space is constructed solely from sampled behaviors that have been screened through the human-evaluated initial latent space. In particular, we clustered our naive latent space using the scikit-learn KMeans function with 15 clusters and default hyperparameters. We visualized the behaviors present at the sample closest to the median of each cluster, and we assigned an acceptance probability to that cluster; if the cluster was synchronized or nearly synchronized, it received an acceptance probability of 0; for nontrivial dynamics involving a single population, we assigned a probability of 0.5; and for nontrivial dynamics involving multiple populations, we assigned a probability of 1.

In the next, “human-aligned” portion of our search, we initialize with the samples from clusters in the initial, naive run, which received an acceptance probability of 1. Our new autoencoder is initialized with weights from the trained autoencoder saved at the end of the naive run. Every time we sample a new behavior, we run it through the old, naive autoencoder. We determine which cluster the new sample belongs to via the KMeans predict method. Based on the acceptance probability of the predicted cluster, that sample is accepted or rejected. If the sample is accepted, the algorithm proceeds normally. If the sample is rejected, a new sample is taken in parameter space. We collect 3200 (accepted) samples following this procedure. The new autoencoder was retrained for 2000 epochs every 400 samples. Details concerning integration of the dynamical system are identical to those presented in Appendix A.

Following human-aligned curiosity search, we construct a latent space of behaviors, and we cluster in that space [Fig. 7(c), left]. We choose representatives of each cluster, and we analyze the behaviors of those representatives by integrating the phase coherence curves of the whole oscillator ensemble, as well as the phase coherence curves for each of the 10 individual populations.

We find a wide variety of behaviors with nontrivial multipopulation dynamics, in accordance with the human intuition with which we aligned our curiosity searcher [Fig. 7(d)]. We can qualitatively identify certain behaviors such as breathing chimeras (behaviors 7, 14), nearly synchronized (behaviors 13, 15), and chiral phases (behavior 11). Many of the behaviors sampled involve multiple populations overlaying in regular (behavior 1) or chaotic patterns (e.g., behaviors 3, 4, 5, 8, 9), aligning with our human-informed scoring criterion. Even behaviors involving a single desynchronized population can display subtle combinations of chiral and chimeric behavior; note, for example, the way in which the overall coherence is never complete in behavior 10, despite the periodic recurrence of coherence in all populations.

## References

[1] Ross TD, Lee HJ, Qu Z, Banks RA, Phillips R, and Thomson M, Controlling organization and forces in active matter through optically defined boundaries, Nature (London) 572, 224 (2019). 31391558 10.1038/s41586-019-1447-1 PMC6719720

[2] Volpe G, Buttinoni I, Vogt D, Kümmerer H-J, and Bechinger C, Microswimmers in patterned environments, Soft Matter 7, 8810 (2011).

[3] Buttinoni I, Volpe G, Kümmel F, Volpe G, and Bechinger C, Active Brownian motion tunable by light, J. Phys.: Condens. Matter 24, 284129 (2012). 22739052 10.1088/0953-8984/24/28/284129

[4] Zhang R, Redford SA, Ruijgrok PV, Kumar N, Mozaffari A, Zemsky S, Dinner AR, Vitelli V, Bryant Z, Gardel ML , Spatiotemporal control of liquid crystal structure and dynamics through activity patterning, Nat. Mater 20, 875 (2021). 33603187 10.1038/s41563-020-00901-4 PMC8404743

[5] Bäuerle T, Fischer A, Speck T, and Bechinger C, Self-organization of active particles by quorum sensing rules, Nat. Commun 9, 1 (2018). 30104679 10.1038/s41467-018-05675-7 PMC6089911

[6] Wang G, Phan TV, Li S, Wombacher M, Qu J, Peng Y, Chen G, Goldman DI, Levin SA, Austin RH , Emergent field-driven robot swarm states, Phys. Rev. Lett 126, 108002 (2021). 33784150 10.1103/PhysRevLett.126.108002

[7] Dai C and Glotzer SC, Efficient phase diagram sampling by active learning, J. Phys. Chem. B 124, 1275 (2020). 31964140 10.1021/acs.jpcb.9b09202

[8] Grizou J, Points LJ, Sharma A, and Cronin L, A curious formulation robot enables the discovery of a novel protocell behavior, Sci. Adv 6, eaay4237 (2020). 32064348 10.1126/sciadv.aay4237 PMC6994213

[9] Jiang Y, Salley D, Sharma A, Keenan G, Mullin M, and Cronin L, An artificial intelligence enabled chemical synthesis robot for exploration and optimization of nanomaterials, Sci. Adv 8, eabo2626 (2022). 36206340 10.1126/sciadv.abo2626 PMC9544322

[10] Hansen N, The CMA evolution strategy: A comparing review, Towards a New Evolutionary Computation: Advances in the Estimation of Distribution Algorithms (Springer, Berlin, Heidelberg, 2006), pp. 75–102.

[11] Whitelam S and Tamblyn I, Neuroevolutionary learning of particles and protocols for self-assembly, Phys. Rev. Lett 127, 018003 (2021). 34270312 10.1103/PhysRevLett.127.018003

[12] Ferguson AL and Brown KA, Data-driven design and autonomous experimentation in soft and biological materials engineering, Annu. Rev. Chem. Biomol. Eng 13, 25 (2022). 35236085 10.1146/annurev-chembioeng-092120-020803

[13] Shmilovich K, Mansbach RA, Sidky H, Dunne OE, Panda SS, Tovar JD, and Ferguson AL, Discovery of self-assembling π-conjugated peptides by active learning-directed coarse-grained molecular simulation, J. Phys. Chem. B 124, 3873 (2020). 32180410 10.1021/acs.jpcb.0c00708

[14] Mohr B, Shmilovich K, Kleinwächter IS, Schneider D, Ferguson AL, and Bereau T, Data-driven discovery of cardiolipin-selective small molecules by computational active learning, Chem. Sci 13, 4498 (2022). 35656132 10.1039/d2sc00116k PMC9019913

[15] Vaddi K, Chiang HT, and Pozzo LD, Autonomous retrosynthesis of gold nanoparticles via spectral shape matching, Digital Discov 1, 502 (2022).

[16] Coli GM, Boattini E, Filion L, and Dijkstra M, Inverse design of soft materials via a deep learning-based evolutionary strategy, Sci. Adv 8, eabj6731 (2022). 35044828 10.1126/sciadv.abj6731 PMC8769546

[17] Carrasquilla J and Melko RG, Machine learning phases of matter, Nat. Phys 13, 431 (2017). 10.1038/s41598-017-09098-0 PMC5562897 28821785

[18] McGibbon RT, Husic BE, and Pande VS, Identification of simple reaction coordinates from complex dynamics, J. Chem. Phys 146, 044109 (2017). 28147508 10.1063/1.4974306 PMC5272828

[19] Van Damme R, Coli GM, Van Roij R, and Dijkstra M, Classifying crystals of rounded tetrahedra and determining their order parameters using dimensionality reduction, ACS Nano 14, 15144 (2020). 33103878 10.1021/acsnano.0c05288 PMC7690044

[20] Gilpin W, Chaos as an interpretable benchmark for forecasting and data-driven modelling, in Thirty-fifth Conference on Neural Information Processing Systems Datasets and Benchmarks Track (Round 2) (2021).

[21] Ricci M, Moriel N, Piran Z, and Nitzan M, Phase2vec: Dynamical systems embedding with a physics-informed convolutional network, arXiv:2212.03857.

[22] Miles C, Samajdar R, Ebadi S, Wang TT, Pichler H, Sachdev S, Lukin MD, Greiner M, Weinberger KQ, and Kim E-A, Machine learning discovery of new phases in programmable quantum simulator snapshots, Phys. Rev. Res 5, 013026 (2023).

[23] Thiem TN, Kooshkbaghi M, Bertalan T, Laing CR, and Kevrekidis IG, Emergent spaces for coupled oscillators, Front. Comput. Neurosci 14, 36 (2020). 32528268 10.3389/fncom.2020.00036 PMC7247828

[24] Dulaney AR and Brady JF, Machine learning for phase behavior in active matter systems, Soft Matter 17, 6808 (2021). 34223598 10.1039/d1sm00266j

[25] Kottmann K, Huembeli P, Lewenstein M, and Acín A, Unsupervised phase discovery with deep anomaly detection, Phys. Rev. Lett 125, 170603 (2020). 33156639 10.1103/PhysRevLett.125.170603

[26] Venderley J, Khemani V, and Kim E-A, Machine learning out-of-equilibrium phases of matter, Phys. Rev. Lett 120, 257204 (2018). 29979078 10.1103/PhysRevLett.120.257204

[27] Oudeyer P-Y, Kaplan F, and Hafner VV, Intrinsic motivation systems for autonomous mental development, IEEE Trans. Evol. Comput 11, 265 (2007).

[28] Baranes A and Oudeyer P-Y, Active learning of inverse models with intrinsically motivated goal exploration in robots, Robot. Auton. Syst 61, 49 (2013).

[29] Reinke C, Etcheverry M, and Oudeyer P-Y, Intrinsically motivated discovery of diverse patterns in self-organizing systems, International Conference on Learning Representations (2020).

[30] Etcheverry M, Moulin-Frier C, Oudeyer P-Y, and Levin M, AI-driven automated discovery tools reveal diverse behavioral competencies of biological networks, eLife 13, RP92683 (2024). 10.7554/eLife.92683 PMC11729405 39804159

[31] Kuramoto Y, Self-entrainment of a population of coupled non-linear oscillators, in International Symposium on Mathematical Problems in Theoretical Physics: January, 1975, Kyoto University, Kyoto/Japan (Springer, Berlin, Heidelberg, 1975), pp. 420–422.

[32] Acebrón JA, Bonilla LL, Vicente CJP, Ritort F, and Spigler R, The Kuramoto model: A simple paradigm for synchronization phenomena, Rev. Mod. Phys 77, 137 (2005).

[33] Kingma DP and Welling M, Auto-encoding variational bayes, arXiv:1312.6114.

[34] Liu Y-H and Van Nieuwenburg EP, Discriminative cooperative networks for detecting phase transitions, Phys. Rev. Lett 120, 176401 (2018). 29756840 10.1103/PhysRevLett.120.176401

[35] Coley CW, Eyke NS, and Jensen KF, Autonomous discovery in the chemical sciences part II: outlook, Angew. Chem., Int. Ed 59, 23414 (2020). 10.1002/anie.201909989 31553509

[36] Wang L, Discovering phase transitions with unsupervised learning, Phys. Rev. B 94, 195105 (2016).

[37] Wetzel SJ, Unsupervised learning of phase transitions: From principal component analysis to variational autoencoders, Phys. Rev. E 96, 022140 (2017). 28950564 10.1103/PhysRevE.96.022140

[38] Rodriguez-Nieva JF and Scheurer MS, Identifying topological order through unsupervised machine learning, Nat. Phys 15, 790 (2019).

[39] Van Nieuwenburg EP, Liu Y-H, and Huber SD, Learning phase transitions by confusion, Nat. Phys 13, 435 (2017).

[40] Prabhakar A and Murphey T, Mechanical intelligence for learning embodied sensor-object relationships, Nat. Commun 13, 4108 (2022). 35840570 10.1038/s41467-022-31795-2 PMC9287329

[41] Abrams DM, Mirollo R, Strogatz SH, and Wiley DA, Solvable model for chimera states of coupled oscillators, Phys. Rev. Lett 101, 084103 (2008). 18764617 10.1103/PhysRevLett.101.084103

[42] Cho YS, Nishikawa T, and Motter AE, Stable chimeras and independently synchronizable clusters, Phys. Rev. Lett 119, 084101 (2017). 28952757 10.1103/PhysRevLett.119.084101

[43] Nicolaou ZG, Eroglu D, and Motter AE, Multifaceted dynamics of Janus oscillator networks, Phys. Rev. X 9, 011017 (2019).

[44] Zhang Y, Nicolaou ZG, Hart JD, Roy R, and Motter AE, Critical switching in globally attractive chimeras, Phys. Rev. X 10, 011044 (2020).

[45] Abrams DM and Strogatz SH, Chimera states for coupled oscillators, Phys. Rev. Lett 93, 174102 (2004). 15525081 10.1103/PhysRevLett.93.174102

[46] Fruchart M, Hanai R, Littlewood PB, and Vitelli V, Non-reciprocal phase transitions, Nature (London) 592, 363 (2021). 33854249 10.1038/s41586-021-03375-9

[47] Ott E and Antonsen TM, Low dimensional behavior of large systems of globally coupled oscillators, Chaos 18, 037113 (2008). 19045487 10.1063/1.2930766

[48] McInnes L, Healy J, and Astels S, hdbscan: Hierarchical density based clustering, J. Open Source Softw 2, 205 (2017).

[49] Moulin-Frier C and Oudeyer P-Y, Curiosity-driven phonetic learning, in 2012 IEEE International Conference on Development and Learning and Epigenetic Robotics (ICDL) (IEEE, Piscataway, NJ, 2012), pp. 1–8.

[50] Gatti G, Brennan M, and Tang B, Some diverse examples of exploiting the beneficial effects of geometric stiffness nonlinearity, Mech. Syst. Sign. Proc 125, 4 (2019).

[51] Tanaka G, Yamane T, Héroux JB, Nakane R, Kanazawa N, Takeda S, Numata H, Nakano D, and Hirose A, Recent advances in physical reservoir computing: A review, Neural Netw 115, 100 (2019). 30981085 10.1016/j.neunet.2019.03.005

[52] Fliess M and Join C, Model-free control, Int. J. Control 86, 2228 (2013).

[53] http://link.aps.org/supplemental/10.1103/PhysRevResearch.6.033052 for additional details.

[54] Etcheverry M, Moulin-Frier C, and Oudeyer P-Y, Hierarchically organized latent modules for exploratory search in morphogenetic systems, in Advances in Neural Information Processing Systems, edited by Larochelle H, Ranzato M, Hadsell R, Balcan MF, and Lin H (Curran Associates, Inc., 2020), Vol. 33, pp. 4846–4859.

